# The NADC30-like PRRSV activates the integrin *α*V subunit to facilitate its entry into Marc-145 cells

**DOI:** 10.1371/journal.pone.0316239

**Published:** 2025-03-27

**Authors:** Chunlin Li, Jin Cui, Hui Zheng, Zhou Sha, Rong Wei, Rui Wu, Bo Ni

**Affiliations:** 1 China Animal Health and Epidemiology Center, Qingdao, China; 2 Heilongjiang Bayi Agricultural University, Daqing, China; 3 Key Laboratory of Animal Biosafety Risk Prevention and Control (South), Ministry of Agriculture and Rural Affairs, Qingdao, People’s Republic of China; 4 Key Laboratory of Animal Biosafety, Qingdao, China; 5 Jiamusi University, Jiamusi, China; 6 Qingdao Key Laboratory of Modern Bioengineering and Animal Disease Research, Qingdao, China; Shanxi University, CHINA

## Abstract

Porcine reproductive and respiratory syndrome virus (PRRSV) is a highly contagious virus that poses a significant threat to the global pig farming industry, resulting in substantial economic losses. However, owing to the high variability of PRRSV and unclear mechanisms of infection, there are currently no effective vaccines or drugs available for its prevention and control. Our previous report revealed that highly pathogenic PRRSV (HP-PRRSV) requires the FAK-PI3K-AKT signaling pathway to facilitate its entry into cells. In this study, we further investigated whether the integrin subunit was involved in the entry process of NADC30-like PRRSV. First, the integrin subunits in Marc-145 cells were characterized by RT-PCR, and 11 of these subunits were identified, nearly all of which interacted with the integrin *α* V and *β*1 subunits to form heterodimers. Western blot analysis revealed that the integrin *α* V subunit was highly expressed in Marc-145 cells, and blocking this subunit with a functional antibody or siRNA significantly attenuated NADC30-like PRRSV entry without affecting virus binding. Moreover, in Marc-145 cells, NADC30-like PRRSV could activate the FAK-PI3K-AKT signaling pathway through the integrin *α* V subunit. Blocking the *α* V subunit significantly inhibited signal transduction and virus entry, and treatment of cells with the PI3K activator greatly reversed this inhibitory effect. Furthermore, the *α* V subunit activator manganese could also enhance NADC30-like PRRSV entry and signal transduction. In conclusion, our results revealed that NADC30-like PRRSV could activate the integrin *α* V subunit and subsequently transduce signals to the FAK-PI3K-AKT signaling pathway to facilitate entry into Marc-145 cells.

## Introduction

Porcine reproductive and respiratory syndrome virus (PRRSV) is a single-stranded RNA virus that belongs to the family Arteriviridae [1]. PRRSV infection usually causes respiratory symptoms, productive failure and immune depression in pig herds, leading to tremendous losses in the pig raising industry worldwide [2]. It has been estimated to cause losses of more than *$*600 million annually in the United States [3]. Despite considerable research, the factors influencing the various stages of PRRSV infection, such as virus entry, replication in host cells, and virus assembly and release, are still poorly understood [4]. NADC30-like PRRSV is a variant of PRRSV that may have been introduced into China from North America in 2013 [5] and was widespread in China in 2014 [6]. This strain is now the main circulating strain of PRRSV in China and has high recombination potential and pathogenicity diversity [7]. Marc-145 is a derivative of the African green monkey kidney cell MA-104 and is often used in virology research and vaccine production because of its high sensitivity to viral infections, particularly PRRSV [8]. Marc-145 cells play crucial roles in understanding the biology and pathogenesis of PRRSV [9].

Integrins are a prominent class of cell surface molecules that play crucial roles in cellular adhesion and signaling. Composed of noncovalently linked *α* and *β* subunits [10], these transmembrane glycoproteins typically have a single membrane-spanning segment and a short cytoplasmic tail [11]. To date, research has identified 18 integrin *α* subunits and 8 *β* subunits, which combine to form over 24 distinct integrin complexes [12]. Predominantly, these heterodimers contain the *α* V or *β*1 subunits, indicating their significant roles in cellular processes [13]. Integrins act as essential transmembrane linkers that connect the extracellular matrix (ECM) to the cytoskeleton, facilitating various cellular functions through biochemical signal transduction across the plasma membrane [14]. ECM binding induces clustering and/or conformational changes in integrins [15], which can elicit cellular signaling events that increase ligand affinity/avidity and promote cytoskeletal rearrangement. Moreover, integrins play important roles in the attachment and entry of various kinds of viruses [16,17]. Notably, integrin subunits are reportedly involved in the infection process of PRRSV, suggesting that integrins are potential targets for antiviral strategies [18]. Understanding the diverse roles of integrins in cellular and viral biology enhances our ability to develop targeted therapeutic interventions in virology.

Integrin assembly facilitates the formation of focal adhesions (FAs), which are crucial for cellular communication and structural integrity [19]. These assemblies recruit signaling molecules, including focal adhesion kinase (FAK), steroid receptor coactivator (Src), and p130 Crk-associated substrate (p130Cas), together with cytoskeletal proteins such as talin, paxillin, and vinculin [20]. These complexes not only strengthen cell-ECM connections but also activate signaling pathways that regulate cell migration, proliferation, and even pathogen infection [19]. FAK is a cytoplasmic tyrosine kinase that is recruited as a participant in focal adhesion dynamics between cell motility and survival. In many cell types, FAK colocalizes with integrins in focal adhesions [21]. The binding of integrins to their extracellular ligands activates FAK and its subsequent tyrosine, serving as a pivotal step in cellular signaling [22–24]. Activated FAK can colocalize and associate with PI3-kinase in cells such as fibroblasts and platelets, which leads to phosphorylation of the p85 subunit of PI3-kinase, demonstrating that PI3-kinase acts as a downstream effector of FAK [25,26]. Phosphorylation of the p 85 subunit of PI3-kinase is a critical step that activates the p110 subunit of PI3-kinase, catalyzing the production of phosphatidylinositol 3,4,5trisphosphate (PIP3), which initiates the PI3K/Akt signaling pathway [27–29]. Our previous study revealed that activation of the FAK-PI3K-AKT-Rac1 signaling pathway during entry is increased in the HP-PRRSV strain [30].

In this study, we used Marc-145 cells to investigate the role of the integrin *α* V subunit, which regulates downstream signaling pathways involved in the cellular entry of NADC30-like PRRSV. These findings indicated that NADC30-like PRRSV induced activation of the integrin pathway before or during entry.

## Materials and methods

### Cells and virus

Marc-145 cells were cultured in Dulbecco’s modified Eagle’s medium (DMEM) (Viva Cell, Italy) supplemented with 10% heatinactivated fetal bovine serum (FBS; OPCEL BS-1101, China.), penicillin (100*U* ∕ *ml* )  and streptomycin  ( 100*μg* ∕ *ml*). Porcine reproductive and respiratory syndrome virus PRRSV (NADC30-like strain) was propagated in Marc-145 cells cultured in DMEM supplemented with 2*%* fetal bovine serum. The virus was purified, titered and then stored at −80∘C until use.

### Chemicals, antibodies and other reagents

Antibodies against FAK and AKT were purchased from Cell Signaling Technology. Antibodies against integrin *α* V, phosphoAKT (S473), phospho-FAK (Y397), and integrin *β*1 were purchased from Abcam, and antibodies against integrin *β*1 were purchased from Abcam and Cell Signaling Technology. A function-blocking antibody against integrin *α* V was purchased from Sigma. Monoclonal antibodies against the PPRSV N protein were purchased from Gene Tex, and PI3K activators were obtained from Santa Cruz and dissolved in DMSO. All the reagents were stored at −20∘C in single-use aliquots. siRNAs designed specifically to knock down integrin *αV* expression were purchased from Sangong along with the corresponding control siRNAs.

### siRNA transfection

Marc-145 cells were grown to 70*%* − 80*%* confluence in cell culture plates and then transiently transfected with small interfering RNA  ( siRNA ) − *αV* using Lipofectamine 3000 (Thermo Fisher) according to the manufacturer’s instructions. The silencing efficiency of the siRNAs was detected via western blot analysis. Scrambled siRNAs were used as negative controls.

### Virus entry inhibition assay

Serum-starved cells transfected with siRNA or preincubated with function-blocking antibodies against integrins (10*μg* ∕ *ml* )  were incubated with NADC 30–like at 4∘C for 1 h to allow virus binding but not internalization. The cells were washed, and internalization was initiated by transferring the cells to 37∘C for 40 min. The medium contained all the inhibitors. The cells were washed with citric acid buffer (40 mM citric acid, 10 mM KCl, and 135 mM NaCl, *pH*3 . 0 )  3 times to inactivate any particles that remained on the cell surface. The cells were then washed three times with PBS to remove the acidic buffer. The internalized virus was detected via both western blotting and real-time PCR.

### Virus binding inhibition assay

Cells transfected with siRNA or preincubated with functionblocking antibodies against integrin *αV* were incubated in DMEM at 37∘C for 1 h. Then, the Marc-145 cells were incubated with NADC30-like PRRSV (MOI = 1) at 4∘C for 1 h to allow virus binding but not internalization. The cells were washed three times with cold PBS to remove the unbound virus particles. The internalized virus was detected via both western blotting and real-time PCR.

### Western blot analysis

Briefly, the cells were washed with PBS three times, freed by scraping, and incubated on ice with Sangong cell lysis buffer (China). The cell lysates were sonicated and then centrifuged at 14 , 000 × *g* for 20 min at 4∘C. The protein concentration was determined using the BCA assay. Equal amounts of protein samples were diluted in 5 ×  SDS-PAGE loading buffer and separated on SDS-PAGE gels. The proteins in the gel were transferred to PVDF membranes, which were then blocked with 5*%* nonfat dry milk in PBST at 4∘C overnight and incubated for 2 h with different primary antibodies. Next, the membrane was incubated for 1 h with the appropriate secondary antibodies. The immunoreactive bands were visualized using an enhanced chemiluminescence system (Vazyme, China).

### Quantitative real-time PCR

Total mRNA was extracted from Marc-145 cells using TRIzol RNA extraction reagent (Takara Bio, Inc., Japan) according to the manufacturer’s instructions. Quantitative real-time PCR was performed using an Evo M-MLV One Step RT-qPCR Kit II (Accurate Biology, China) with the following procedures: reverse transcription at 42∘C for 6 min, predenaturation at 95∘C for 10 min, denaturation at 95∘C for 10 s, and annealing and extension at 60∘C for 20 s for a total of 45 cycles. Each sample was run in triplicate. The relative amount of mRNA for the target gene was normalized to that of actin mRNA in the same sample. The primer and probe sequences used are shown in Table 1.

**Table 1:** Primers and probes for qRT–PCR.

Gene	Primer sequence (5’ to 3’)
N	Forward: TTGTGTCTGTCGTCGATCCAG
	Reverse: AAACTCCACAGTGTAACTTATCCTC
	Probe: (FAM) CGCTGGAACTTGTGCCCTGTCA (Eclipse)
	Forward: TGACTGACTACCTCATGAAGATCC
Actin	Reverse: TCTCCTTAATGTCACGCACGATT
	Probe: (FAM) CGGCTACAGCTTCACCACCACGGC (Eclipse)

### Integrin characterization by RT-PCR

Integrin characterization via RT-PCR was performed as reported previously [31]. In brief, total mRNA was extracted from Marc-145 cells using TRIzol RNA extraction reagent (Takara Bio Inc., Japan) according to the manufacturer’s instructions. Reverse transcription reactions were performed using a HiScriptIV 1st Strand cDNA Synthesis Kit II (+gDNA wiper) (Vazyme, China). RT-PCR amplification was performed using 2 ×  Phanta Flash Master Mix(Dye Plus) (Vazyme, China) with the following procedure: denaturation at 95∘C for 15 min, followed by 35 to 45 cycles of 95∘C for 45 s, 55∘C for 45 s, and 72∘C for 10 s. The sequences of the primers used are listed in Table 2.

**Table 2:** Primers.

Gene target	Forward	Reverse	Size
*α*1	aatgagcctggagcctatca	tatacacggctcctccgtga	198 bp
*α*2	actttgttgctggtgctcct	caagagcacatcggtaatgg	179 bp
*α*3	aagccaagtctgagact	gtagtattggtcccgagtct	657 bp
*α*4	agcaccatcagagaggaagg	gcagaatcagaccgaaaagc	383 bp
*α*5	atcagagcaagagccggata	ttagtgtcccggagatgagg	388 bp
*α*6	ggccttatgaagttggtgga	tgccttgctggttcatgtag	375 bp
*α*7	gcatcaagagcttcggctac	acagactcggtcatgctgg	410 bp
*α*8	cgcaggtggaaataagagg	tctctggaggaaggtgtgg	530 bp
*α*9	agaggaggagagggaactgc	cccagacaggtggcttgtat	436 bp
*α*10	atggctccaacagcatctacc	aggaaagagctgggatctcg	439 bp
*α*11	gctccaacagcatctaccc	ggtcactggcgatgtatttg	459 bp
*α* V	tgctgatacaacaggcttgc	tccatctctgactgctggtg	420 bp
*β*1	gtggaggaaatggtgtttgc	gtctgcccttggaacttgg	184 bp
*β*2	caagctggctgaaaacaaca	actgctcctggatgcactct	347 bp
*β*3	agatgcgaaagctcaccagt	ccgtcattaggctggacaat	391 bp
*β*4	gccttcactttgagcactcc	ctgctgtactcgctttgcag	383 bp
*β*5	agcagcacccatgtcttgc	gaagttgctggtgagcttcc	276 bp
*β*6	gactccggaaacattctcca	ctgacagtcgcagttgcatt	306 bp
*β*7	agcaatggcctctacagtcgcagc	gcttggagagaaacccagaaagtc	392 bp
*β*8	tccatgtgctgtctttgacc	tgtcatcaccagcagcaatc	179 bp

## Results

### Integrin subunit characterization

**Fig 1:**
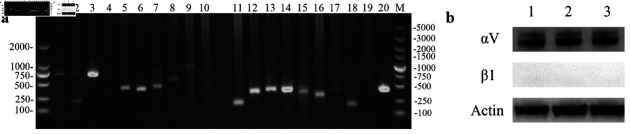
Integrin subunit characterization in Marc145 cells. (a) Integrin subunit characterization by RT-PCR. Lane 1: *α*1; Lane 2: *α*2; Lane 3: *α*3; Lane 4: *α*4; Lane 5: *α*5; Lane 6: *α*6; Lane 7: *α*7; Lane 8: *α*8; Lane 9: *α*9; Lane 10: *α*10; Lane 11: *β*1; Lane 12: *β*2; Lane 13: *β*3; Lane 14: *β*4; Lane 15: *β*5; Lane 16: *β*6; Lane 17 : *β*7; Lane 18 : *β*8; Lane 19: *α*11; Lane 20: *α* V; M: marker. (b) -3 western blot analysis of integrin *αV* and the *β*1 subunit in Marc- 145 cells.

It is well known that 18 integrin *α* subunits and 8 integrin *β* subunits can assemble into more than 24 different integrin complexes in cells. First, we characterized the integrin subunits expressed in the Marc-145 cell line. The mRNA levels of the integrin subunits were tested via RT-PCR with 26 pairs of integrin subunit-specific primers. Amplicons of the integrin *αV* , *α*3 , *α*5 , *α*6, *α*7 , *β*1 , *β*2 , *β*3 , *β*4 , *β*6, and *β*8 subunits were detected (Fig 1a). Since nearly all of these subunits interacted with the integrin *αV* and *β*1 subunits to form heterodimers, we further verified whether the *αV* and *β*1 subunits were expressed in Marc-145 cells. Western blotting was performed, and the *α* V subunit was strongly detected; however, the *β*1 subunit was not detected despite the use of two kinds of commercial antibodies (Fig 1b).

### NADC30-like PRRSV entry requires the integrin *α* V subunit

**Fig 2:**
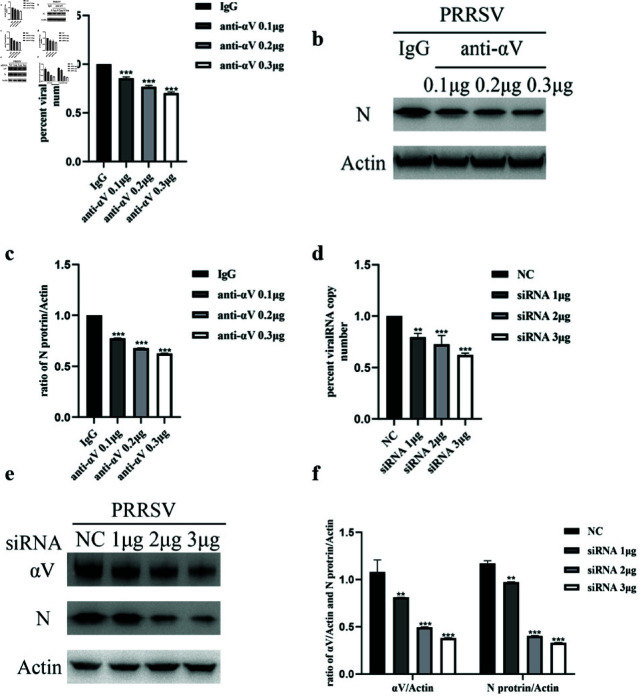
NADC30-like PRRSV entry requires the integrin *αV* subunit. An integrin *αV* neutralization antibody blocks virus entry. (a) qRT-PCR or (b) western blotting. siRNA-mediated knockdown of the integrin *αV* subunit significantly reduces virus entry, as shown by (d) qRT-PCR and (e) western blotting. (c)(f) Ratios of N or integrin protein to actin corresponding to (b) and (e) are shown.

Since we detected the expression of *αV* in Marc-145 cells, we further verified whether the integrin *α* V subunit participated in the NADC30-like PRRSV entry process. First, we incubated the cells with a function-blocking antibody against *αV* integrin to determine whether the *αV*-specific antibody could block NADC30-like PRRSV entry. The internal virus concentration was measured via qRT-PCR and western blot analysis. qRT-PCR revealed that the integrin *α* V neutralizing antibody inhibited NADC30-like PRRSV entry in a dose-dependent manner (Fig 2a), which was also confirmed by western blot analysis (Fig 2b and 2c). To avoid the nonspecific effect of neutralization antibodies, siRNAs targeting integrin *α* V were used. The siRNA construct specifically reduced the total amount of integrin *α* V protein (Fig 2d and 2e), and the amount of PRRSV structural protein inside the cell was also reduced, suggesting that virus entry was inhibited. These results were further verified via qRT-PCR and revealed that siRNA targeting the integrin *αV* subunit could inhibit NADC30-like PRRSV entry in a dose-dependent manner (Fig 2f).

### The integrin *αV*subunit is not involved in the binding of NADC30-like PRRSV

**Fig 3:**
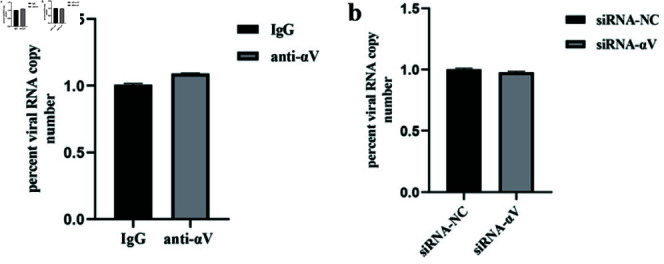
The integrin *αV* subunit is not involved in the binding step of the NADC30-like PRRSV. Blocking the integrin *α* V subunit with (*a*) a neutralizing antibody or (*b*) siRNA–*αV* does not influence virus binding.

The possibility that the integrin *αV* subunit regulated PRRSV entry by affecting the binding step was ruled out. We further tested the possible role of integrin *α* V in the binding of NADC30-like PRRSV to target cells. Neither the neutralizing antibody nor the siRNA influenced virus binding (Fig 3a and 3b).

### NADC30-like PRRSV entry activates the FAK-PI3K-AKT pathway through the integrin *αV* subunit

**Fig 4:**
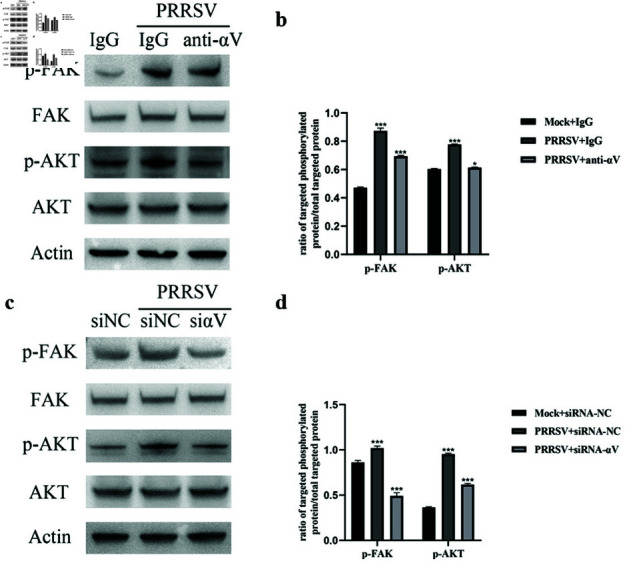
NADC30-like PRRSV activates the FAK-PI3K-AKT pathway through integrin *αV.* NADC30-like PRRSV entry can activate the FAK-PI3K-AKT pathway, and blocking the integrin *αV* subunit with (a) neutralizing antibodies or (c) siRNA–*αV* decreases the phosphorylation of FAK and AKT. (b)(d) The ratios of p-FAK and p -AKT to total protein in (a) and (c) are shown.

To verify whether NADC30-like PRRSV initiated the activation of the FAK-PI3K-AKT signaling cascade through the integrin *αn* subunit during the entry process, Marc-145 cells pretreated with integrin *α* V blocking antibody or siRNA were infected with NADC30-like PRRSV, and the cells were harvested 15 min p.i. Since AKT is a key downstream intermediate in PI3K-dependent signaling, we selected FAK and AKT as detection targets to verify whether the signaling pathway was activated. The results indicated that FAK and AKT phosphorylation were significantly elevated in infected cells, indicating that, similar to HP-PRRSV, NADC30-like PRRSV could activate the FAK signaling pathway. Blocking the function of integrin *α* V with a neutralizing antibody (Fig 4a and 4b) and siRNA reduced the activation of FAK and AKT in NADC30-like PRRSV-infected cells (Fig 4c and 4d). These results showed that NADC30-like PRRSV required the integrin *α* V subunit to activate FAK-PI3K-AKT during entry.

### Integrin *αV* subunit regulates NADC30-like PRRSV entry through the FAK-PI3K-AKT pathway

**Fig 5:**
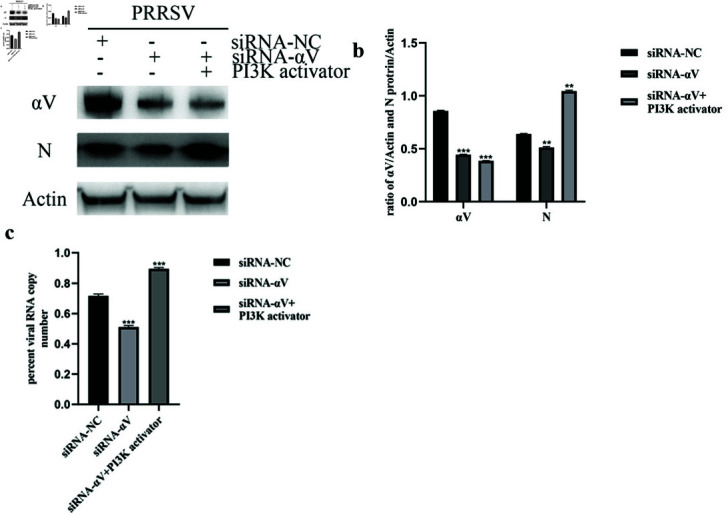
The integrin *αV* subunit regulates NADC30-like PRRSV entry through the FAK-PI3K-AKT pathway. (a) The PI3K activator significantly increases virus entry in integrin *α* V knockdown MARC-145 cells, as shown by both western blotting and (c) real-time RT-PCR. (b) The ratio of N protein to actin in (a) is shown.

To confirm that integrin *α* V regulated NADC30-like PRRSV entry through the FAK-PI3K-AKT pathway, we first knocked down the integrin *α* V subunit with siRNA to block signaling and virus entry. In the next step, we treated *α* V subunit knockdown cells with a PI3K activator to determine whether the activation of downstream effectors could reverse the inhibitory effect on virus entry caused by the loss of upstream signals. Western blot and qRT-PCR results revealed that integrin *α* V knockdown reduced NADC30-like PRRSV entry, and the PI3K activator strongly reversed this inhibitory effect in Marc-145 cells (Fig 5a, 5b, and 5c). These results showed that when integrin *α* V acted upstream, NADC30-like PRRSV stimulated the FAK-PI3K-AKT pathway through the integrin *α* V subunit during entry.

### Activation of integrin by Mn^2+^ can increase signal transduction and NADC30-like PRRSV entry

**Fig 6:**
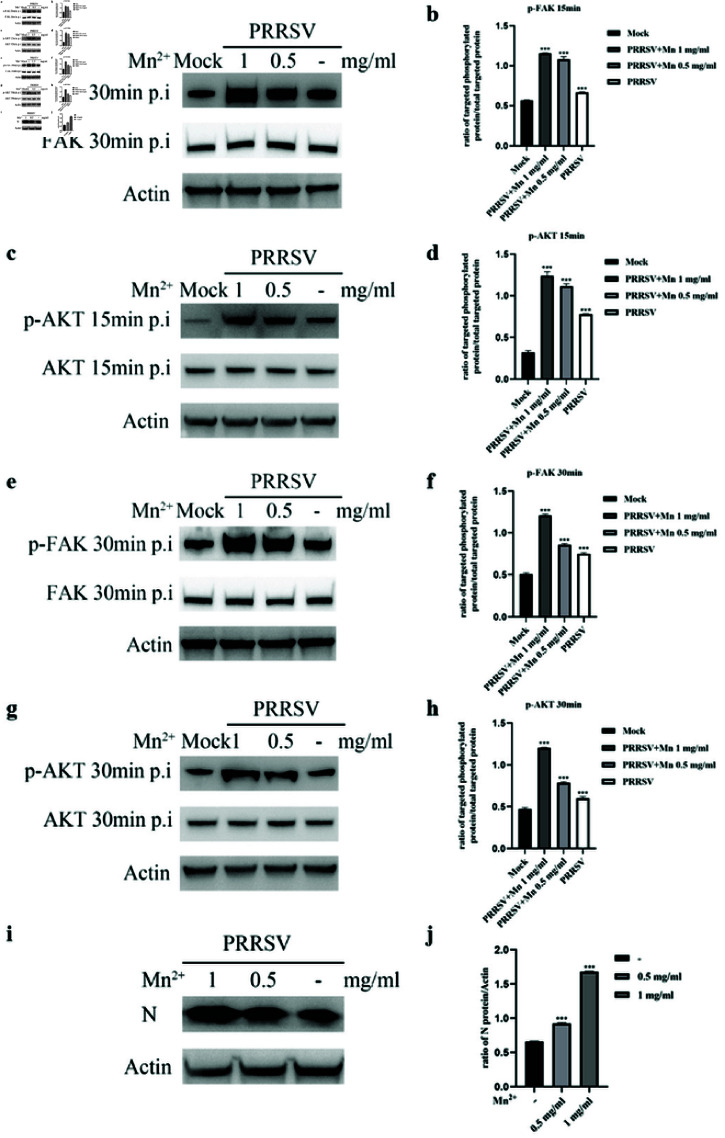
Activation of integrin by Mn^2+^ can enhance signal transduction and NADC30-like PRRSV entry. Mn^2+^ increases FAK phosphorylation at 15 min p.i. (a) and 30 min p.i. (e). Mn^2+^ increases the phosphorylation of AKT at 15*minp* . *i*. (c) and 30*minp* . *i*. (g). The ratios of p-FAK and p-AKT to total protein in (a), (c), (e), and (g) are shown in (b), (d), (f), and (h), respectively. (i) Mn^2+^ enhances NADC30-like PRRSV entry. (j) The ratio of N protein to actin in (i) is shown.

Mn^2+^ can induce the extension of integrins in the absence of ligands and partially activate integrins [32–34]. We investigated whether Mn^2+^ treatment could enhance NADC30-like PRRSV entry and related signal transduction. Marc-145 cells were incubated with NADC30-like PRRSV for 1 h in the presence or absence of Mn^2+^ before being transferred to 37∘C. Marc- 145 cells were harvested at 15 min and 30 min postinfection for western blot analysis. As expected, Mn^2+^ increased FAK and AKT phosphorylation (Fig 6a–6h). Mn^2+^ treatment also increased virus entry (Fig 6i and 6j). These results further verified the above findings that NADC30-like PRRSV utilized integrins to control the FAK-PI3K-AKT signaling pathway to facilitate its entry.

## Discussion

PRRSV is a major pathogen in the swine industry that causes significant economic losses worldwide. It can cause symptoms in affected pigs, such as respiratory difficulties, weight loss, poor growth performance, and high fever [34]. It is associated with high morbidity and mortality rates, causing substantial economic losses to the pig farming industry worldwide. The development of effective vaccines and drugs against PRRSV has been challenging because of the high genetic variability and complex infection mechanisms of this virus. As an RNA virus, PRRSV has a high mutation rate due to the lack of proofreading activity in its RNAdependent RNA polymerase. This intrinsic characteristic facilitates rapid genetic diversification within and between viral genotypes over time [24]. Epidemiological investigations have shown that multiple PRRSV strains are now prevalent in China, including HPPRRSV, CH-1a PRRSV, NADC30-like PRRSV, and NADC34-like PRRSV. Among them, NADC30-like PRRSV is the main circulating PRRSV strain [35]. Thus, in this study, we used NADC30-like PRRSV for our research.

The entry mechanism of PRRSV into host cells is a critical aspect of its infection process. Initially, the virus attaches to the cell surface through interactions with heparan sulfate, which provides a low-affinity binding site, facilitating preliminary anchoring of the virus to the cell membrane [36]. Subsequently, the viral GP5/M protein complex exhibits increased specificity for binding to the N-terminal domain of CD169 (sialoadhesin) on susceptible cells. This binding event is crucial, as it stimulates receptor-mediated, clathrin-dependent endocytosis, resulting in internalization of the virus into the host cell via clathrin-coated vesicles. The viral particles are then transported to early endosomes, where the acidic environment, along with interactions with CD163, promotes uncoating of the viral genome, facilitating its release into the cytoplasm [37]. The CD163 receptor plays a pivotal role in the infection process in vivo, acting as a critical facilitator of successful PRRSV infection. Moreover, additional receptors might be involved in this complex entry process, particularly in the context of Marc-145 cells, where simian vimentin and CD151 have been implicated as potential contributors to PRRSV entry and infection [38]. Our previous study revealed that HP-PRRSV can stimulate phosphorylation of the FAK-PI3K-AKT pathway to precisely regulate the cytoskeleton to promote its endocytosis. However, the upstream signaling molecules are still unknown. Integrins are ubiquitously expressed in virtually all cells. Integrin clusters recruit signaling molecules such as talin, FAK, Src and PI3K to form focal contacts that regulate outside-in signaling to manipulate important cellular activities [39]. Therefore, we hypothesized that integrins might be upstream signaling molecules that activate the FAK-PI3K-AKT signaling pathway to facilitate PRRSV entry.

Since there are 26 kinds of integrin subunits in different cell types, we first characterized the integrin subunits in Marc-145 cells via RT-PCR, and 11 subunits, namely, *αV* , *α*3 , *α*5 , *α*6 , *α*7 , *β*1, *β*2 , *β*3 , *β*4 , *β*6, and *β*8, were identified. Nearly all of these proteins, except for *β*2, which is expressed mainly in leukocytes, can interact with the *α* V and *β*1 subunits to form a complex [40]. Western blot results revealed that the *α* V subunit was highly expressed in Marc-145 cells, and the *β*1 subunit was not detected using antibodies from two reputable companies, Abcam and Cell Signaling. Therefore, we focused our research on the *α* V subunit, and further studies were performed to investigate whether integrin *α* V was involved in NADC30-like PRRSV entry.

First, we investigated whether NADC30-like PRRSV entry required the integrin *α* V subunit. After blocking integrin activity with blocking antibodies and siRNAs (control IgG and siRNA-NC were used to avoid nonspecific effects), the amount of internalized virus protein and virus mRNA significantly decreased in a dosedependent manner. These results showed that NADC30-like PRRSV required the integrin *αV* subunit for entry. Moreover, similar to HP-PRRSV, NADC30-like PRRSV was able to activate the FAK-PI3K-AKT signaling pathway during entry. When Marc145 cells were incubated with NADC30-like PRRSV, FAK and AKT phosphorylation significantly increased. In contrast, blocking the *α* V subunit with neutralizing antibodies and siRNAs significantly inhibited signal transduction and virus entry. These results suggested that NADC30-like PRRSV activated the FAK-PI3K-AKT signaling pathway through the integrin *α* V subunit. However, we could not completely inhibit signal transduction by blocking the activity of integrins, which we hypothesized might indicate that the integrin *α* V subunit was not the only molecule involved in signal transduction. To confirm that integrin *α* V regulated NADC30-like PRRSV entry through the FAK-PI3K-AKT pathway, we treated siRNA- *α* V-transfected Marc-145 cells with a PI3K activator to determine whether downstream signaling molecule activators compensated for the loss of upstream signaling. As expected, the PI3K activator strongly reversed this inhibitory effect. Moreover, the *α* V subunit activator manganese was able to enhance NADC30-like PRRSV entry and signal transduction. PRRSV exhibits strong tropism for cells of the monocyte/macrophage lineage, which are critical components of the host immune defense system [41], and the data obtained using Marc-145 cells may not fully reflect the situation in vivo. However, since Marc-145 is a well-established cellular model of PRRSV infection [42], our results may provide insights and scientific data for studying the molecular mechanisms of NADC30-like PRRSV entry.

## Conclusions

Our results revealed that NADC30-like PRRSV could activate the integrin *αV* subunit and subsequently transduce signals to the FAK-PI3K-AKT signaling pathway to facilitate entry into Marc-145 cells.
